# Pre-delivery prediction of vaginal delivery in late-onset foetal growth restriction: development and internal validation of a model with decision curve analysis and bedside calculator

**DOI:** 10.1007/s00404-026-08462-8

**Published:** 2026-06-09

**Authors:** Manal Massalha, Misgav Rottenstreich, Bryon DeFrance, Kinga Malinowski, Jon F. Barrett, Eran Ashwal

**Affiliations:** 1 Ruth and Bruce Rappaport Faculty of Medicine, Technion - Israel Institute of Technology, Haifa, Israel; 2 Department of Obstetrics and Gynecology, McMaster University, Hamilton, Canada; 3 Department of Obstetrics & Gynecology, Shaare Zedek Medical Center, Hebrew University School of Medicine, 91031 Jerusalem, Israel; 4 Department of Health Research, Evidence, and Impact, McMaster University, Hamilton, Canada

**Keywords:** Foetal growth restriction, Cerebroplacental ratio, Prediction model, Vaginal delivery, Induction of labour, Decision curve analysis

## Abstract

**Purpose:**

Foetal growth restriction (FGR) complicates 5–10% of pregnancies and is a major contributor to perinatal morbidity and mortality. In late-onset FGR, induction of labour is recommended; however, intrapartum caesarean rates remain high (20–40%). Accurate counselling regarding the likelihood of vaginal delivery is essential, yet no validated prediction model exists for this population. We aim to develop and internally validate a pre-delivery prediction model for vaginal delivery in patients with late-onset FGR undergoing a trial of labour, and to assess its clinical utility.

**Methods:**

This retrospective cohort study included singleton pregnancies with late-onset FGR managed at a tertiary centre (2017–2024). Eligible patients had no prior caesarean delivery, underwent attempted labour, and had complete Doppler assessment within 7 days of delivery. Logistic regression models were constructed using clinical and Doppler variables. Internal validation was performed using bootstrap resampling, and clinical utility was assessed with decision curve analysis.

**Results:**

Among 477 patients, 378 (79.2%) achieved vaginal delivery. Nulliparity (OR 0.22, 95% CI 0.12–0.38; *p* < 0.001), pre-pregnancy BMI (OR 0.93 per kg/m^2^, 95% CI 0.90–0.96; *p *< 0.001), abnormal cerebroplacental ratio (OR 0.47, 95% CI 0.28–0.78; *p* = 0.004), and pre-gestational diabetes (OR 0.28, 95% CI 0.10–0.78; *p* = 0.015) were independently associated with lower odds of vaginal delivery. The model demonstrated moderate discrimination (AUC 0.74, 95% CI 0.70–0.80) and good calibration, with net benefit above a 55% threshold.

**Conclusion:**

This four-variable model provides individualised prediction of vaginal delivery in late-onset FGR. External validation is required before clinical implementation.

**Supplementary Information:**

The online version contains supplementary material available at 10.1007/s00404-026-08462-8.

## What does this study add to the clinical work?

**Table:** 

A model incorporating nulliparity, pre-pregnancy body mass index, abnormal cerebroplacental ratio Doppler (<5th centile), and pre-gestational diabetes mellitus predicted vaginal delivery with a corrected AUC of 0.741 and demonstrated clinical utility from the 55% threshold probability onward. To our knowledge, this is the first internally validated prediction model with decision curve analysis and bedside calculator developed specifically for Delphi-defined late-onset FGR.	

## Introduction

Foetal growth restriction (FGR) complicates 5–10% of pregnancies and remains a leading cause of perinatal morbidity and mortality. In 2016, the Delphi consensus established standardised diagnostic criteria for late-onset FGR that integrate estimated foetal weight or abdominal circumference thresholds with Doppler indices and growth trajectory [1]. These criteria have improved diagnostic precision and facilitated international comparisons; however, the management of delivery in late-onset FGR remains a source of clinical uncertainty.

When delivery is indicated in late-onset FGR, induction of labour is the predominant approach. Caesarean delivery (CD) rates following induction in this population are reported between 20 and 40%, considerably higher than in the general obstetric population [2]. Pre-delivery counselling regarding the likelihood of vaginal delivery (VD) is an integral component of informed consent; however, clinicians currently lack a validated tool to individualise this estimate. Existing clinical prediction models for induction outcomes have been developed predominantly in unselected or small-for-gestational-age (SGA) populations, and their applicability to Delphi-defined FGR is uncertain [3, 4].

Few prediction models have been developed specifically for delivery outcome in FGR. Villalain et al. developed a bootstrap-validated model in 201 pregnancies with late-onset FGR undergoing induction at or beyond 37 weeks, incorporating cerebroplacental ratio (CPR), pre-eclampsia, and parity as predictors [5]. That model was limited by a small sample, absence of clinical utility assessment, and lack of a bedside tool for counselling. Other models in the broader FGR and SGA literature have relied on intrapartum variables such as Bishop score after cervical ripening, used birthweight-defined SGA populations rather than Delphi criteria [1], or reported unadjusted associations without formal validation [3, 4]. To our knowledge, no prior prediction model in this population has incorporated decision curve analysis or provided an implemented calculator for use at the point of delivery planning, although a formal systematic search for all existing models was not performed.

Doppler velocimetry of the foetal circulation is central to the surveillance and delivery planning.

The objective of this study was to develop and internally validate a pre-delivery prediction model for VD in patients with late-onset FGR who attempted labour. Secondary objectives were to compare alternative Doppler assessments (CPR versus umbilical artery pulsatility index [UA-PI] and middle cerebral artery pulsatility index [MCA-PI] entered separately), to evaluate clinical utility through decision curve analysis, and to provide an interactive bedside calculator for clinical application.

## Methods

### Study design and setting

This was a retrospective cohort study conducted at a single tertiary centre. The study population was drawn from a prospectively maintained obstetric database encompassing all singleton pregnancies managed between January 2017 and December 2024. The study was approved by the local Research Ethics Board (#2024–15003). Reporting followed the Transparent Reporting of a multivariable prediction model for Individual Prognosis or Diagnosis (TRIPOD) guidelines for model development and internal validation [6].

### Population

Eligible pregnancies met all of the following criteria: (1) singleton gestation; (2) late-onset FGR diagnosed at or beyond 32 + 0 weeks of gestation [1]; (3) attempted VD (spontaneous or induced labour); and (4) complete Doppler assessment (UA-PI, MCA-PI, and CPR) obtained within 7 days of delivery.

Late-onset FGR was classified by one of two pathways [1]. The solitary pathway required an estimated foetal weight (EFW) or abdominal circumference (AC) below the 3rd centile on any scan at or beyond 32 weeks. The contributory pathway required at least two of three criteria: (1) EFW or AC below the 10th centile; (2) a decline of more than 50 centile points in EFW or AC between any two scans during the pregnancy; and (3) CPR below the 5th centile or UA-PI above the 95th centile for gestational age. A single qualifying scan was sufficient for classification.

Pregnancies with prior CD undergoing a trial of labour after caesarean section (TOLAC) and those delivered by prelabour caesarean section were excluded.

### Outcome

The primary outcome was VD, defined as any delivery completed vaginally (spontaneous or operative vaginal delivery). The comparator was CD performed after the onset of labour.

### Candidate predictors

All candidate predictors were restricted to information available before the onset of labour, to ensure the model could be applied at the point of pre-induction counselling. Predictors were selected based on prior literature and clinical reasoning and comprised three domains: maternal characteristics, foetal Doppler indices, and pre-existing medical conditions.

Candidate predictors were selected on the basis of clinical plausibility and prior evidence of association with delivery mode. Maternal characteristics included nulliparity, pre-pregnancy body mass index (BMI) (continuous, kg/m^2^), maternal age (continuous, years), and gestational age at delivery (continuous, weeks). Pre-gestational diabetes mellitus (type 1 or type 2) was included as a binary variable.

Hypertensive disorders (i.e. chronic hypertension, gestational hypertension, and pre-eclampsia) and amniotic fluid volume (maximum vertical pocket) were considered but not included as candidate predictors, as both were judged to lie on the causal pathway to FGR rather than acting as independent determinants of delivery mode.

Doppler indices were obtained from the last pre-delivery ultrasound scan within 7 days of delivery, reflecting the standard weekly surveillance interval for late-onset FGR at the study institution. All three indices (UA-PI, MCA-PI, CPR) were required from the same scan. Doppler parameters were classified as binary variables using published gestational-age-specific reference ranges: UA-PI above the 95th centile (Acharya et al. [7]), MCA-PI below the 5th centile (Bahlmann et al. [8]), and CPR below the 5th centile (Ebbing et al. [9]). Doppler indices were entered as binary variables using established gestational-age-specific thresholds, consistent with their use in clinical practice and the Delphi diagnostic criteria [1]. A supplementary analysis compared binary, continuous, and log-transformed Doppler parameterisations (Table S1). EFW was estimated using the Hadlock formula [10], and the corresponding centile was derived from Hadlock’s gestational-age-specific reference ranges [11]. EFW centile was included in the clinical models as a continuous variable.

### Statistical analysis

#### Sample size

The minimum sample size for model development was estimated following the framework of Riley et al. [12]. For eight-candidate predictor parameters (the maximum number evaluated across all development models), an anticipated outcome prevalence of approximately 20% (CD), and a target calibration slope shrinkage factor of 0.9 or greater, the minimum required sample size was estimated at approximately 320 pregnancies with at least 64 outcome events.

#### Model development

The analytical cohort comprised pregnancies with complete data for all candidate predictors across all four models (*n* = 477). This common complete-case set was used throughout to ensure consistent denominators across all analyses.

Normality of continuous variables was assessed by the Shapiro–Wilk test. Normally distributed variables were summarised as mean ± standard deviation and compared between groups using the independent-samples t-test; non-normally distributed variables were summarised as median (interquartile range) and compared using the Mann–Whitney U test. Categorical variables were compared using the *χ*^2^ test or the Fisher exact test when any expected cell count was less than 5.

Four multivariable logistic regression models were developed to predict VD: Model 1 included CPR (binary, < 5th centile) with clinical variables (nulliparous, BMI, maternal age, pre-gestational diabetes, gestational age at delivery, and EFW centile; 7 predictors). Model 2 included UA-PI (binary, > 95th centile) and MCA-PI (binary, < 5th centile) with the same clinical variables (8 predictors). Model 3 retained CPR (binary) alongside only those clinical variables that were statistically significant (*p* < 0.05) in Model 1. Model 4 retained UA-PI and MCA-PI (binary) alongside the significant clinical variables from Model 2; both Doppler components were retained for conceptual coherence, as CPR is the mathematical ratio of MCA-PI to UA-PI.

The primary model was selected on the basis of parsimony, EPV, and calibration performance. A head-to-head comparison of Doppler indices was performed by sequentially adding each index to a base model containing parity, BMI, and pre-gestational diabetes mellitus, with improvement assessed by the likelihood-ratio Chi-squared test.

#### Internal validation

Internal validation was performed using bootstrap resampling with 1000 iterations. In each iteration, a model was fitted on a bootstrap sample drawn with replacement and then applied to the original dataset. The optimism in apparent performance was estimated as the mean difference between the bootstrap-sample AUC and the original-sample AUC across all iterations. The optimism-corrected AUC was calculated by subtracting this mean optimism from the apparent AUC. Ninety-five percent confidence intervals for the corrected AUC were derived from the bootstrap distribution.

#### Model performance

Discrimination was assessed by the area under the receiver operating characteristic curve (AUC). Calibration was evaluated by the calibration slope, the Hosmer–Lemeshow goodness-of-fit test with 10 groups, and a calibration plot of observed versus predicted probabilities by decile. Overall predictive accuracy was quantified by the Brier score.

Clinical utility was assessed by decision curve analysis, which plots the net benefit of the model against the strategies of attempting VD in all patients or performing CD in all patients, across a range of threshold probabilities [13]. The threshold probability represents the minimum predicted probability of VD at which the expected benefits of a labour attempt are judged to outweigh the consequences of a failed trial resulting in intrapartum CD. Because the study cohort comprised only patients who attempted labour, the decision curve quantifies the net benefit of using the model versus the default strategies of attempting VD in all patients or in none, within this population.

#### Sub-analyses

Pre-specified sub-analyses were performed by onset of labour. In the induction-of-labour subgroup, Model 3 was refitted with bootstrap validation as described above, and the full-cohort model coefficients were applied without refitting to assess transportability. The same transportability assessment was planned for the spontaneous-labour subgroup; refitting was contingent on adequate sample size (EPV > = 10).

### Missing data

A complete-case analysis was performed. The common complete-case set required non-missing values for all predictors across all four models. Characteristics of excluded pregnancies were compared with those included to assess whether missingness was plausibly at random. Multiple imputation was not performed, given the low overall proportion of missing data (33/510, 6.5%) and the absence of strong auxiliary variables that would improve imputation quality beyond the predictors themselves. The potential impact of this decision is addressed in the Limitations.

### Software

All analyses were performed in Python (version 3.12) using statsmodels (version 0.14) for logistic regression, scikit-learn (version 1.4) for discrimination metrics, and SciPy (version 1.12) for the Hosmer–Lemeshow test. An interactive bedside calculator was developed as a web-based tool to facilitate clinical application of the primary model.

## Results

### Study population

**Fig. 1:**
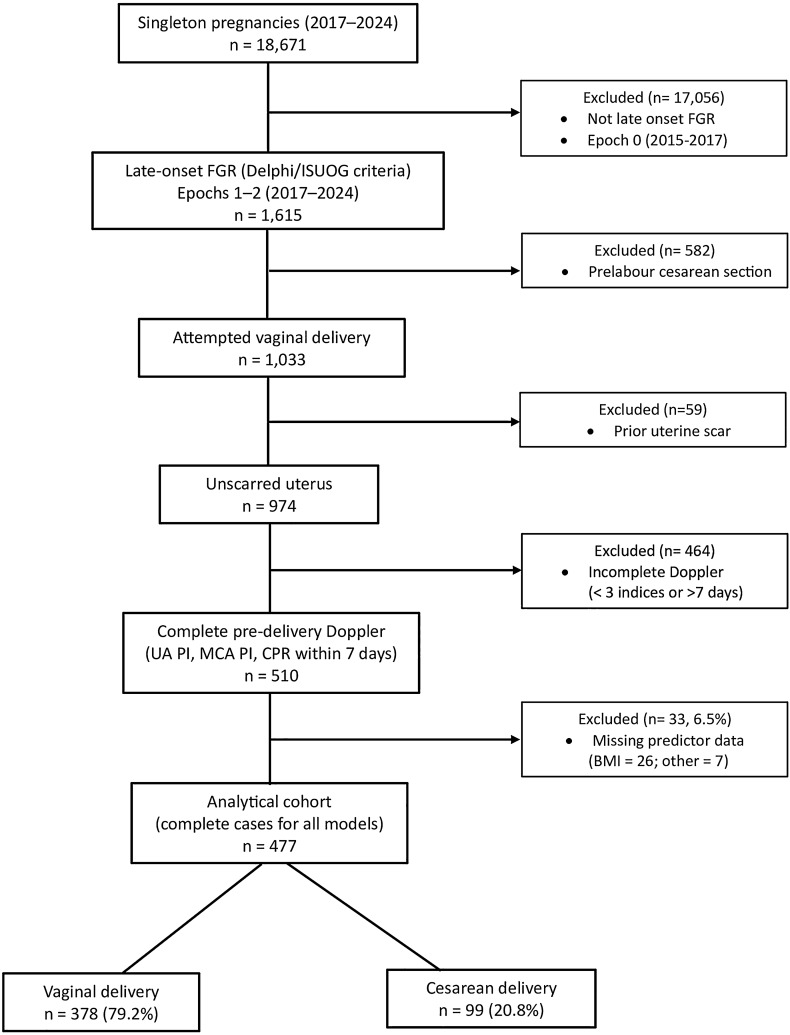
Flow diagram of study population. From 18,671 singleton pregnancies (2017–2024), sequential application of exclusion criteria yielded 510 eligible pregnancies with Delphi-defined late-onset foetal growth restriction, attempted labour, unscarred uterus, and complete pre-delivery Doppler assessment within 7 days of delivery. After exclusion of 33 pregnancies with missing predictor data, the analytical cohort comprised 477 pregnancies

During the study period (2017–2024), 18,671 singleton pregnancies were managed at the study institution. After the application of exclusion criteria, 1615 pregnancies were classified as late-onset FGR according to the Delphi consensus definition [1]. Of these, 1033 attempted VD. Complete Doppler assessment (UA-PI, MCA-PI, and CPR from the same scan within 7 days of delivery) was available for 510 pregnancies. Of the 510 eligible pregnancies, 33 (6.5%) were excluded for missing data in at least one predictor. The analytical cohort comprised 477 pregnancies (Fig. 1).

Among the 477 pregnancies, 378 (79.2%) achieved VD and 99 (20.8%) were delivered by CD after the onset of labour. Baseline characteristics of the cohort are presented in Table 1. Among the Doppler indices, 279 (58.5%) patients had an abnormal CPR below the 5th centile, 148 (31.0%) had UA-PI above the 95th centile, and 92 (19.3%) had MCA-PI below the 5th centile.

**Table 1:** Pre-delivery characteristics and perinatal outcomes of the analytical cohort by mode of delivery (*n* = 477)

	Total (*n* = 477)	VD (*n* = 378)	CS (*n* = 99)	*p* value
Pre-delivery characteristics				
Maternal age (years)	30.0 (26.0–35.0)	31.0 (27.0–35.0)	29.0 (26.0–34.0)	0.221
Pre-pregnancy BMI (kg/m^2^)	23.9 (21.3–28.4)	23.5 (21.0–26.9)	27.0 (22.4–30.5)	< 0.001
Nulliparous	270 (56.6)	191 (50.5)	79 (79.8)	< 0.001
Smoking at first visit	75 (15.7)	55 (14.6)	20 (20.2)	0.169
Assisted conception	79 (16.6)	64 (16.9)	15 (15.2)	0.672
Pre-gestational diabetes	18 (3.8)	9 (2.4)	9 (9.1)	0.002
Chronic hypertension	12 (2.5)	7 (1.9)	5 (5.1)	0.070
Pre-eclampsia	21 (4.4)	12 (3.2)	9 (9.1)	0.011
Gestational hypertension	26 (5.5)	19 (5.0)	7 (7.1)	0.425
Gestational diabetes	54 (11.3)	42 (11.1)	12 (12.1)	0.778
Ultrasound and Doppler parameters				
EFW (g)^†^	2370 ± 419	2384 ± 407	2320 ± 461	0.246
EFW centile^†^	4.3 (1.5–9.2)	4.5 (1.6–9.3)	3.6 (1.2–8.4)	0.323
AC centile^†^	4.1 (0.9–15.2)	4.4 (1.1–15.9)	2.7 (0.2–10.9)	0.050
UA-PI > 95th centile	148 (31.0)	108 (28.6)	40 (40.4)	0.023
MCA-PI < 5th centile	92 (19.3)	64 (16.9)	28 (28.3)	0.011
CPR < 5th centile	279 (58.5)	207 (54.8)	72 (72.7)	0.001
Oligohydramnios	24 (5.0)	17 (4.5)	7 (7.1)	0.297
Delivery and neonatal outcomes				
Induction of labour	402 (84.3)	315 (83.3)	87 (87.9)	0.269
GA at delivery (weeks)	37.1 (36.4–38.0)	37.3 (36.4–38.1)	36.9 (36.3–37.9)	0.045
Birthweight (g)	2473 ± 428	2503 ± 417	2354 ± 450	0.001
Birthweight centile (Hadlock)	1.1 (0.1–5.9)	1.5 (0.2–7.2)	0.4 (0.0–2.4)	< 0.001
Operative vaginal delivery	41 (8.6)	36 (9.5)	5 (5.1)	0.157
Neonatal sex (male)	252 (53.1)	203 (54.0)	49 (49.5)	0.425
Apgar 5 min < 7^§^	8 (1.7)	5 (1.3)	3 (3.0)	0.373
NICU transfer	145 (30.4)	100 (26.5)	45 (45.5)	< 0.001

Normality of continuous variables was assessed by the Shapiro–Wilk test. Normally distributed variables (EFW, AC, birthweight) are presented as mean ± SD and compared using the independent-samples *t*-test; non-normally distributed variables are presented as median (interquartile range) and compared using the Mann–Whitney *U* test. Categorical variables are presented as *n* (%) and compared using the *χ*^2^ test or Fisher exact test when any expected cell count was less than 5. All Doppler measurements from the pre-delivery scan within 7 days

*BMI* body mass index, *CPR* cerebroplacental ratio, *CS* caesarean section, *EFW* estimated foetal weight, *GA* gestational age, *MCA* middle cerebral artery, *MVP* maximal vertical pocket, *NICU* neonatal intensive care unit, *PI* pulsatility index, *UA* umbilical artery, *VD* vaginal delivery

^†^EFW and AC from last biometry scan within 14 days of delivery (Dopplers weekly, biometry fortnightly)

^§^Apgar 5 min available in 474 patients (99.4%); 3 missing

### Prediction model development

Four logistic regression models were developed (Table 2). Model 3 retained nulliparity, BMI, abnormal CPR, and pre-gestational diabetes mellitus (4 predictors). Model 4 retained nulliparity, BMI, UA-PI, MCA-PI, and pre-gestational diabetes mellitus (5 predictors). In Model 3, the designated primary model, four predictors were independently associated with VD: nulliparity (odds ratio [OR] 0.218; 95% confidence interval [CI] 0.124–0.383; *p* < 0.001), pre-pregnancy BMI (OR 0.930 per kg/m^2^; 95% CI 0.898–0.963; *p* < 0.001), abnormal CPR below the 5th centile (OR 0.466; 95% CI 0.278–0.781; *p* = 0.004), and pre-gestational diabetes mellitus (OR 0.275; 95% CI 0.097–0.779; *p* = 0.015). The apparent area under the receiver operating characteristic curve (AUC) was 0.749. After bootstrap validation with 1000 resamples, the optimism-corrected AUC was 0.741 (95% CI 0.697–0.800), and the optimism-corrected AUC after bootstrap validation with 1,000 resamples was 0.741 (95% CI 0.697–0.800). The calibration slope was 0.946 and the Brier score was 0.141. The Hosmer–Lemeshow goodness-of-fit test indicated adequate calibration (*p* = 0.367).

**Table 2:** Multivariable logistic regression models for prediction of vaginal delivery in late-onset foetal growth restriction

	Model 1	Model 2	Model 3^†^	Model 4
	CPR + clinical	UA/MCA + clinical	CPR + parsimonious	UA/MCA + parsimonious
Predictor–OR (95% CI)				
Nulliparous	0.222 (0.124–0.397)	0.227 (0.127–0.406)	0.218 (0.124–0.383)	0.223 (0.127–0.393)
BMI, per kg/m^2^	0.927 (0.894–0.961)	0.927 (0.895–0.961)	0.930 (0.898–0.963)	0.930 (0.899–0.963)
Pre-gestational DM	0.246 (0.077–0.780)	0.258 (0.080–0.831)	0.275 (0.097–0.779)	0.274 (0.094–0.801)
Maternal age, per year	1.005 (0.961–1.051)	1.004 (0.960–1.051)	–	–
GA at delivery, per week	1.109 (0.931–1.322)	1.142 (0.959–1.359)	–	–
EFW centile (Hadlock)	1.006 (0.991–1.022)	1.006 (0.990–1.022)	–	–
CPR < 5th centile	0.488 (0.288–0.826)	–	0.466 (0.278–0.781)	–
UA-PI > 95th centile	–	0.783 (0.460–1.334)	–	0.769 (0.462–1.280)
MCA-PI < 5th centile	–	0.553 (0.314–0.973)	–	0.538 (0.307–0.942)
*p*-values				
Nulliparous	< 0.001	< 0.001	< 0.001	< 0.001
BMI	< 0.001	< 0.001	< 0.001	< 0.001
Pre-gestational DM	0.017	0.023	0.015	0.018
Maternal age	0.840	0.847	–	–
GA at delivery	0.247	0.136	–	–
EFW centile	0.422	0.485	–	–
CPR < 5th	0.008	–	0.004	–
UA-PI > 95th	–	0.368	–	0.312
MCA-PI < 5th	–	0.040	–	0.030
Model performance				
No. of predictors	7	8	4	5
EPV	14.1	12.4	24.8	19.8
AUC, apparent	0.753	0.759	0.749	0.756
AUC, corrected	0.734	0.735	0.741	0.744
95% CI	0.700–0.802	0.703–0.811	0.697–0.800	0.705–0.804
Calibration slope	0.897	0.872	0.946	0.929
Brier score	0.141	0.141	0.141	0.141
AIC	435.3	439.0	431.4	435.9
BIC	468.6	476.5	452.2	460.9

*AIC* Akaike information criterion, *AUC* area under the receiver operating characteristic curve, *BIC* Bayesian information criterion, *BMI *body mass index, *CI* = confidence interval, *CPR* cerebroplacental ratio, *DM* diabetes mellitus, *EFW* estimated foetal weight, *GA* gestational age, *MCA* middle cerebral artery, *OR* odds ratio, *PI* pulsatility index, *EPV* events per variable, *UA *umbilical artery

^†^Primary model. All models fitted on *n* = 477 complete cases (vaginal delivery 378 [79.2%], caesarean section 99 [20.8%]). Bootstrap internal validation: 1000 resamples

–Predictor not included in model

Model 3 was designated as the primary model. It had the fewest significant predictors among the reduced models, the highest EPV (24.8), and the highest calibration slope among the four models. The clinical models (Models 1 and 2), which additionally included maternal age, gestational age, and EFW centile, did not improve discrimination (corrected AUC 0.734 and 0.735, respectively) and showed greater optimism (calibration slopes 0.897 and 0.872, respectively).

The analytical cohort exceeded the pre-specified minimum sample size requirement, with events per variable of 12.4 for the most complex model (Model 2, 8 predictors) and 24.8 for the primary model (Model 3, 4 predictors).

### Head-to-head Doppler comparison

Sequential addition of each Doppler index to a base model containing parity, BMI, and pre-gestational diabetes mellitus (AUC 0.731) was used to compare the incremental value of individual indices. Addition of CPR (abnormal < 5th centile) yielded a statistically significant improvement (ΔAUC + 0.018; *p* = 0.004), as did MCA-PI (abnormal < 5th centile; ΔAUC + 0.023; *p* = 0.018). UA-PI (abnormal > 95th centile) alone did not reach significance (ΔAUC + 0.008; *p* = 0.170). The combined addition of both UA-PI and MCA-PI (ΔAUC + 0.025; *p* = 0.041) did not meaningfully exceed the improvement achieved by CPR alone. Continuous and log-transformed Doppler parameterisations yielded modestly higher apparent AUCs than their binary counterparts (e.g. log CPR: AUC 0.761 versus binary CPR: AUC 0.749), but continuous MCA-PI did not significantly improve the base model (*p* = 0.147), consistent with extreme right skewness of its distribution (skewness 17.4). Binary classification was retained for the primary models (Table S1).

### Decision curve analysis

**Fig. 2:**
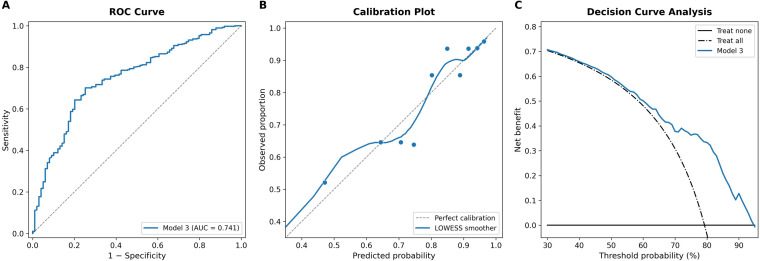
Performance of the primary model (Model 3). **A** Receiver operating characteristic curve (optimism-corrected AUC 0.741; 95% CI 0.697–0.800). **B** Calibration plot of observed versus predicted vaginal delivery rates by decile. **C** Decision curve analysis comparing Model 3 with treat-all (attempt vaginal delivery in all) and treat-none strategies across threshold probabilities from 30 to 95%

**Fig. 3:**
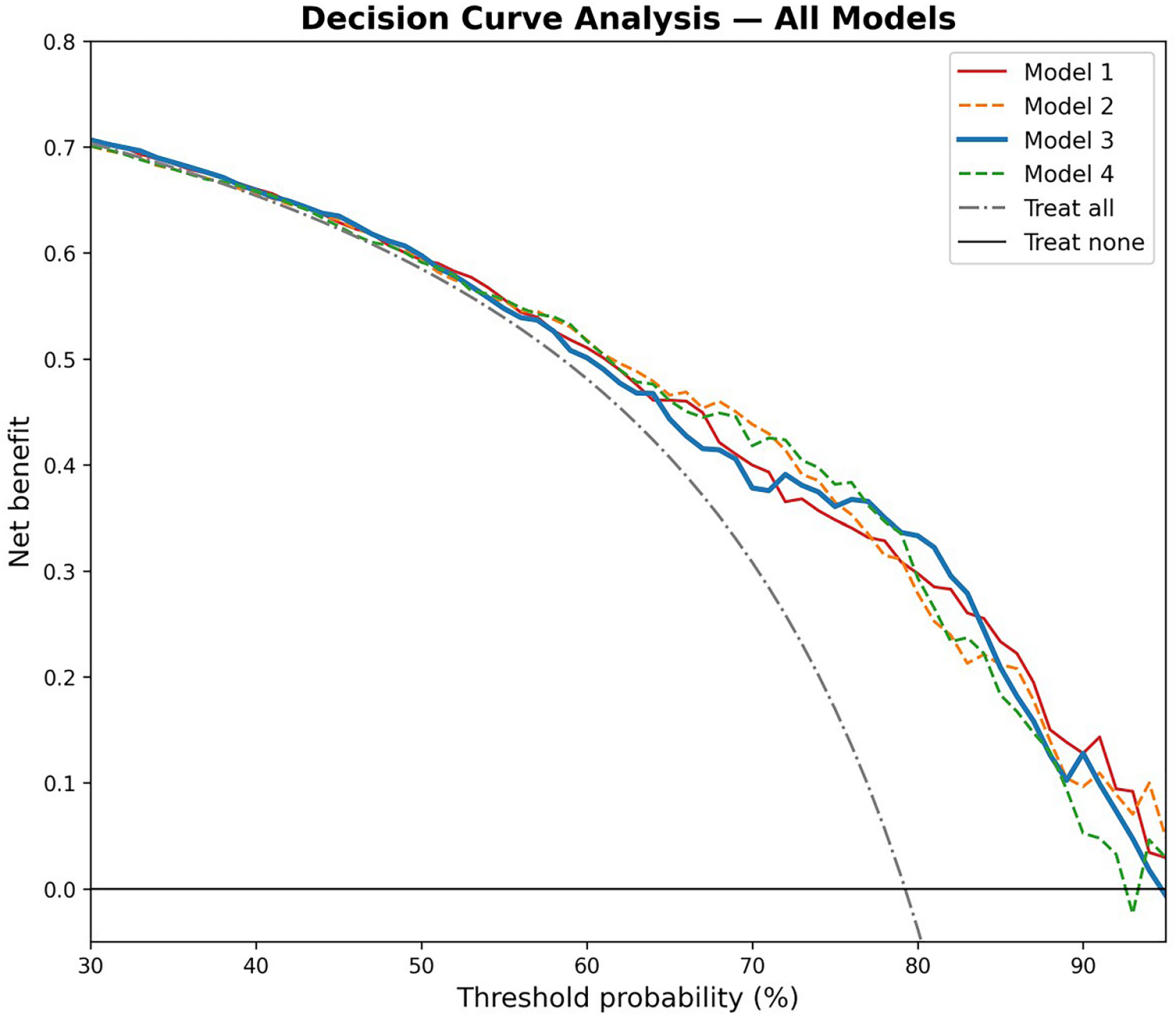
Decision curve analysis comparing all four prediction models. Net benefit is plotted against threshold probability. All four models produce virtually identical decision curves, consistent with their similar discrimination

Decision curve analysis demonstrated that, among patients who attempted labour, Model 3 provided net benefit above the treat-all strategy from approximately a 55% threshold probability onward (Fig. 2). At clinically relevant thresholds of 60%, 70%, and 80%, the model yielded net benefit advantages of + 0.020, + 0.079, and + 0.390 over the treat-all approach, respectively. All four models produced virtually identical decision curves, consistent with their similar discrimination (Fig. 3).

### Sub-analyses by onset of labour

Among the 477 patients, 402 (84.3%) underwent induction of labour and 75 (15.7%) entered spontaneous labour (Table 3). The VD rate was 78.4% in the induction subgroup and 84.0% in the spontaneous-labour subgroup.

**Table 3:** Sub-analysis of Model 3 by onset of labour

	Full cohort	Induction of labour	Spontaneous labour
	(*n* = 477)	(*n* = 402)	(*n* = 75)
Outcome			
Vaginal delivery, *n* (%)	378 (79.2)	315 (78.4)	63 (84.0)
Caesarean section, *n* (%)	99 (20.8)	87 (21.6)	12 (16.0)
Model 3 (refitted)–OR (95% CI)			
Nulliparous	0.218 (0.124–0.383)	0.251 (0.139–0.454)	–
BMI, per kg/m^2^	0.930 (0.898–0.963)	0.931 (0.896–0.968)	–
CPR < 5th centile	0.466 (0.278–0.781)	0.498 (0.291–0.853)	–
Pre-gestational DM	0.275 (0.097–0.779)	0.324 (0.103–1.025)	–
*p*-values			
Nulliparous	< 0.001	< 0.001	–
BMI	< 0.001	< 0.001	–
CPR < 5th	0.004	0.011	–
Pre-gestational DM	0.015	0.055	–
Performance (refitted model)			
AUC, apparent	0.749	0.735	–
AUC, corrected	0.741	0.725	–
95% CI	0.697–0.800	0.668–0.784	–
Calibration slope	0.946	0.935	–
Brier score	0.141	0.149	–
H–L *p*-value	0.367	0.272	–
Full-cohort model applied			
AUC	–	0.734	0.844
95% CI	–	–	0.697–0.960
Mean predicted VD, %	–	78.4	80.2
Mean observed VD, %	–	78.4	84.0

Model 3: nulliparous + BMI + CPR < 5th centile + pre-gestational DM. Refitted = model re-estimated on the sub-cohort with bootstrap internal validation (1000 resamples). Full-cohort model applied = coefficients from the primary cohort (*n* = 477) applied without refitting

*H–L* Hosmer–Lemeshow goodness-of-fit test. Other abbreviations as in Table 2

–Not applicable. The spontaneous-labour sub-cohort (*n* = 75, 12 CS events) had insufficient events for stable model refitting (EPV = 3.0); only transportability of the full-cohort model is reported

Model 3 was refitted in the induction subgroup. Three of the four predictors remained significant: nulliparity (OR 0.251; 95% CI 0.139–0.454; *p* < 0.001), BMI (OR 0.931; 95% CI 0.896–0.968; *p* < 0.001), and abnormal CPR (OR 0.498; 95% CI 0.291–0.853; *p* = 0.011). Pre-gestational diabetes mellitus was of borderline significance (OR 0.324; 95% CI 0.103–1.025; *p* = 0.055), which may reflect limited power in this subgroup. The optimism-corrected AUC was 0.725 (95% CI 0.668–0.784) with a calibration slope of 0.935, a Brier score of 0.149, and a Hosmer–Lemeshow *p* = 0.272. When the full-cohort model coefficients were applied to the induction subgroup without refitting, the AUC was 0.734, confirming transportability.

The spontaneous-labour subgroup was too small for stable model refitting (75 patients, 12 CD, EPV = 3.0). The full-cohort model applied to this subgroup produced an AUC of 0.844 (95% CI 0.697–0.960). These results are reported descriptively and are not intended to support model redevelopment or independent application in the spontaneous-labour population.

### Bedside calculator

An interactive web-based calculator was developed from the primary model equation: logit(P) = 4.810 + (− 1.525 × nulliparous) + (− 0.073 × BMI) + (− 0.763 × CPR abnormal) + (− 1.293 × pre-gestational DM). The calculator accepts four inputs (parity, BMI, CPR status, and pre-gestational diabetes status) and returns the estimated probability of VD with a 95% confidence interval. For example, a nulliparous patient with a BMI of 30 kg/m^2^ and abnormal CPR but without pre-gestational diabetes has a predicted probability of vaginal delivery of 58% (95% CI 48–65%), whereas a multiparous patient with a BMI of 25 kg/m^2^ and normal CPR without pre-gestational diabetes has a predicted probability of 95% (95% CI 91–97%). In patients with pre-gestational diabetes, these estimates decrease to 28% and 85%, respectively, reflecting the independent adverse effect of pre-existing diabetes on labour outcome. The tool is intended for use at the point of pre-delivery counselling; the 95% confidence intervals displayed by the calculator are derived from the Wald standard errors of the linear predictor [Supplementary Material].

## Discussion

In this retrospective cohort study of 477 patients with Delphi-defined late-onset FGR who attempted labour, a parsimonious four-variable model incorporating nulliparity, pre-pregnancy BMI, abnormal CPR (< 5th centile), and pre-gestational diabetes mellitus predicted VD with moderate discrimination (optimism-corrected AUC 0.741; 95% CI 0.697–0.800) and adequate calibration. The model demonstrated clinical utility in decision curve analysis. Performance was preserved in the induction-of-labour subgroup (corrected AUC 0.725). An interactive bedside calculator was developed.

### Comparison with existing literature

To our knowledge, this is the first internally validated prediction model for vaginal delivery applied specifically to a Delphi-defined late-onset FGR population [1]. The only prior model meeting comparable eligibility criteria was developed by Villalain et al. in 201 pregnancies with late-onset FGR induced at or beyond 37 weeks, in which CPR, pre-eclampsia, and parity were identified as predictors of VD with bootstrap validation [5]. The present study extends that work in several respects: a larger cohort (*n* = 477), formal model comparison across four candidate specifications, head-to-head Doppler comparison, decision curve analysis, and a bedside calculator. Other models in the broader FGR and SGA literature have included intrapartum variables such as Bishop score after cervical ripening [14] or were developed in SGA-only populations without Delphi criteria [3, 4], limiting their applicability to the clinical scenario addressed here.

The AUC of 0.741 falls within the range reported for prediction models of labour outcome in related populations. Kalafat et al. reported an antenatal-only AUC of 0.69 in SGA pregnancies at term, rising to 0.75 when intrapartum variables were added [4]. Kolli et al. developed logistic regression and machine learning models for caesarean prediction in patients with morbid obesity (BMI ≥ 40 kg/m^2^), reporting AUCs of approximately 0.80 using variables available at admission [15]. Gabbai et al. reported 85% sensitivity and 70% specificity for an intrapartum caesarean prediction score in patients with obesity (*n* = 5663), though that model incorporated intrapartum variables not available before delivery [16]. The present model, restricted to pre-delivery inputs, achieves comparable discrimination without requiring information that is unavailable at the point of counselling.

### Cerebroplacental ratio as a predictor of delivery mode

The finding that abnormal CPR independently predicted CD (OR 0.466; 95% CI 0.278–0.781; *p* = 0.004) is biologically plausible and consistent with accumulating evidence. A low CPR reflects foetal cerebral redistribution in the setting of placental insufficiency and is associated with reduced tolerance of uterine contractions [17]. A systematic review of 16 observational studies encompassing 14,823 patients confirmed that a low CPR was consistently associated with a higher rate of emergency caesarean for intrapartum foetal compromise, though individual predictive accuracy was moderate [18]. The RATIO37 trial (*n* ≈ 11,000), the largest randomised evaluation of CPR-based management at term to date, reported that the addition of the CPR to standard growth assessment did not reduce perinatal mortality but significantly reduced severe neonatal morbidity [19]. These data support the clinical relevance of CPR assessment near delivery, while underscoring that its value may be greatest when integrated into a multivariate framework rather than used as a standalone screening test.

In the present head-to-head comparison, CPR provided a statistically significant improvement over a base model of parity, BMI, and pre-gestational diabetes (ΔAUC + 0.018; *p* = 0.003), as did MCA-PI (ΔAUC + 0.023; *p* = 0.020), whereas UA-PI did not reach significance (ΔAUC + 0.008; *p* = 0.173). The combined entry of UA-PI and MCA-PI did not meaningfully surpass the improvement achieved by CPR alone. This finding is consistent with the theoretical advantage of the CPR as an integrative measure of both placental resistance and cerebral vasodilation, and aligns with the individual participant data meta-analysis by Vollgraff Heidweiller-Schreurs et al. (*n* = 18,731), which found that CPR provided similar prognostic accuracy to UA-PI alone for a composite adverse perinatal outcome [20]. The present study differs by focussing on the specific outcome of delivery mode, in which CPR may be more directly informative than UA-PI given its relationship with foetal intrapartum tolerance.

### Pre-gestational diabetes mellitus

Pre-gestational diabetes mellitus was associated with CD (OR 0.275; 95% CI 0.097–0.779; *p* = 0.015), with a VD rate of 50% among 18 affected patients compared with 80% in those without. The wide confidence interval reflects the small number of affected patients, and this estimate should be regarded as preliminary. Inclusion in the final model was based on the statistical significance in the development cohort and the biological plausibility of the association, but the coefficient may be unstable and will require confirmation on external validation. Patients with pre-gestational diabetes and late-onset FGR presented a distinctive clinical profile: higher BMI (29.6 versus 25.1 kg/m^2^), higher EFW centiles (40th versus 9th centile), and more frequent abnormal UA-PI (72% versus 29%). The relatively higher EFW centiles in this subgroup suggest that FGR was diagnosed predominantly via the contributory pathway (Doppler deterioration or centile crossing) rather than by absolute smallness, a pattern consistent with severe placental disease overriding the macrosomic tendency of diabetic pregnancies. The combination of altered placental function, myometrial effects of diabetes, and higher BMI provides a plausible mechanistic basis for the observed association with CD. Pre-gestational diabetes was of borderline significance in the induction-of-labour subgroup (OR 0.324; *p* = 0.055), which may reflect reduced statistical power given only 15 affected patients in that subgroup. Whether the effect size observed in the full cohort is reproducible remains an open question for external validation. Given the small number of affected patients, this association should be considered hypothesis-generating pending confirmation in an external cohort. Type 1 and type 2 diabetes were analysed together as a single binary variable owing to the small number of affected patients; whether these subtypes exert differential effects on labour outcome in FGR warrants investigation in a larger validation cohort.

### Nulliparity and BMI as predictors

Nulliparity was the strongest predictor in the model (OR 0.218), consistent with the well-established relationship between parity and labour outcome. The association between increasing BMI and CD (OR 0.930 per kg/m^2^ for VD) is concordant with large cohort studies demonstrating that maternal obesity independently increases the risk of CD following induction [21, 22]. The mechanism is thought to involve reduced myometrial contractility, slower cervical dilation, and increased soft tissue resistance [23]. Continuous modelling of BMI was chosen over binary classification at 30 kg/m^2^ because the relationship with delivery mode is approximately linear per unit, and dichotomisation would have discarded information, as demonstrated consistently in the prediction modelling literature [24].

### Clinical utility and the bedside calculator

The decision curve analysis demonstrated that the model provided net benefit above the treat-all strategy from the 55% threshold onward. This threshold range is clinically meaningful in the context of shared decision-making for patients with late-onset FGR in whom labour is being considered. A patient with a predicted VD probability of 55–70% falls within a range where the decision curve analysis suggests potential clinical usefulness of the model. In this range, the benefits of VD (shorter recovery, avoidance of surgical morbidity, preserved future reproductive options) must be weighed against the risks of a failed labour trial (emergency caesarean, foetal distress, prolonged labour); the model may assist in structuring that conversation but does not prescribe a specific threshold for action. Above this threshold, the model identifies a subgroup of patients in whom attempting VD yields more benefit than the default strategy of attempting delivery in all patients, effectively selecting those most likely to succeed. The provision of a bedside calculator translates the model coefficients into a tool that can be used during pre-delivery counselling without requiring statistical computation. No previous prediction model in FGR has provided a comparable implemented tool.

The near-identical decision curves across all four models suggest that the incremental information from additional predictors (age, gestational age, EFW centile) does not translate into meaningfully different clinical recommendations. The base model of nulliparity, BMI, and pre-gestational diabetes alone (AUC 0.731) achieves discrimination only 0.010 AUC units below the primary model. Although the addition of CPR was statistically significant (*p *= 0.003) and biologically motivated, the clinical utility improvement is modest. The selection of Model 3 for implementation is supported by its parsimony and the biological rationale for CPR in FGR, but the base model could serve as a simplified alternative in settings where Doppler data are unavailable.

The sub-analysis by onset of labour confirmed that the model performs adequately in the induction subgroup (corrected AUC 0.725). The transportability of the full-cohort coefficients to this subgroup (AUC 0.734) supports the use of a single model for counselling regardless of planned onset of labour. Future validation studies should aim to enrol sufficient numbers of spontaneous-labour patients to assess model performance in this subgroup independently, as the clinical decision-making context may differ from that of planned induction.

### Strengths

This study has several strengths. First, the population was defined using the Delphi consensus criteria for late-onset FGR [1], rather than SGA or birthweight proxies. Second, all predictors were restricted to information available before the onset of labour, making the model applicable at the actual decision point. Third, model development followed TRIPOD guidelines [7] with bootstrap internal validation, calibration assessment, and decision curve analysis. Fourth, the pre-specified model comparison across four specifications, with head-to-head Doppler testing, provides a transparent rationale for predictor selection. Fifth, the bedside calculator and decision curve analysis address the translational gap between prediction modelling and clinical implementation. Sixth, the sample size of 477 pregnancies with 99 events exceeded the minimum recommended by the Riley framework for the full set of eight-candidate parameters considered during development [12].

### Strengths and limitations

This study has several notable strengths. The model was developed using a well-characterised clinical cohort drawn from a prospectively maintained obstetric database, ensuring high data fidelity. The final model is parsimonious, incorporating only routinely available clinical and sonographic variables, making it readily implementable in standard obstetric practice without requiring additional investigations. The inclusion of a simplified base model (parity, BMI, and pre-gestational diabetes; AUC 0.731) further broadens applicability to settings where complete Doppler surveillance within the delivery window is not feasible. The transparent reporting of model coefficients and calibration supports external validation and potential recalibration across different clinical populations.

Several limitations should also be acknowledged. First, this was a single-centre retrospective study, and external validation in independent populations is required before clinical adoption can be recommended. Second, the requirement for complete Doppler assessment within 7 days of delivery excluded 47.6% of otherwise eligible pregnancies. This reflects a restriction of the target population rather than a missing-data problem: these patients did not undergo the requisite scan within the specified window. Because the interval between the last surveillance scan and delivery varies in clinical practice, the model is applicable only to patients presenting with a recent complete Doppler assessment. Where this is unavailable, the base model may serve as a simplified alternative, though it was not formally validated. Third, the complete-case analysis excluded 33 pregnancies (6.5%) with missing predictor data. Excluded patients had a lower vaginal delivery rate, were older, and delivered at earlier gestational ages, indicating that missingness was not completely at random. Any resulting bias is likely conservative, and the model may slightly underestimate caesarean risk in borderline cases. Multiple imputation was not pursued given the small proportion of missing data and the absence of strong auxiliary variables. Fourth, cervical status (Bishop score) was not incorporated, as it was not uniformly documented before labour onset. Omitting this variable actually enhances the model’s generalisability, since cervical assessment is not standardised in timing and varies considerably across settings. Fifth, the cohort caesarean rate (20.8%) is lower than the 30–40% reported in some FGR induction series, which may reflect institutional practice, population characteristics, or the inclusion of spontaneous-labour pregnancies; performance in higher caesarean rate settings remains to be established. Finally, the number of patients with pre-gestational diabetes was small (*n* = 18), yielding a wide confidence interval for the corresponding odds ratio. While the association is biologically plausible, the coefficient may be unstable at this sample size, and recalibration of the diabetes coefficient may be warranted in populations with a different prevalence of this condition.

### Implications and future directions

The model addresses an unmet clinical need: individualised pre-delivery counselling for patients with late-onset FGR in whom a labour attempt is being considered. It is intended for patients already selected as candidates for a labour attempt; the model does not predict who should attempt labour, but rather, among those in whom VD is being considered, who is most likely to succeed. The four required inputs, parity, BMI, CPR status, and pre-gestational diabetes status, are routinely available at the time of delivery planning, imposing no additional investigational burden.

Future work should prioritise external validation in independent populations. The distinction between reproducibility and transportability is relevant here [25]. Given the relatively low prevalence of Delphi-defined FGR in unselected populations, retrospective validation using existing databases with prospective Doppler surveillance data may offer the most feasible initial design; a mixed retrospective-prospective approach could also be considered, with a minimum target of approximately 100 caesarean events. Additionally, investigation of additional biomarkers, such as placental growth factor (PlGF), which has shown promise for prediction of intrapartum foetal compromise [26], could further improve model discrimination.

## Conclusion

A four-variable pre-delivery prediction model incorporating nulliparity, pre-pregnancy BMI, abnormal CPR, and pre-gestational diabetes mellitus provides moderate discrimination and adequate calibration for vaginal delivery in patients with late-onset FGR. The model demonstrates clinical utility on decision curve analysis from the 55% threshold onward and has been translated into a bedside calculator. External validation in independent populations is needed before clinical implementation.

## Supplementary Information

Below is the link to the electronic supplementary material.

Supplementary file1 (DOCX 18 KB)

## Data Availability

The clinical data that support the findings of this study are derived from routinely collected patient records at McMaster University and are not publicly available due to patient privacy and institutional confidentiality constraints. De-identified data may be made available upon reasonable request to the corresponding author, subject to approval by the institutional review board and data governance committee.
