# Supplementary material for: Pre-delivery prediction of vaginal delivery in late-onset foetal growth restriction: development and internal validation of a model with decision curve analysis and bedside calculator
Source: Arch Gynecol Obstet. 2026 Jun 9;313(1):251. doi: 10.1007/s00404-026-08462-8 (PMC13473075; doi:10.1007/s00404-026-08462-8)
Supplement: Supplementary file 1 [file 404_2026_8462_MOESM1_ESM.docx]

**Table S1.** Comparison of binary, continuous, and log-transformed Doppler parameterisations for prediction of vaginal delivery. All models include nulliparity, pre-pregnancy BMI, and pre-gestational diabetes mellitus as base predictors (n = 477).

| **Model** | **Doppler OR (95% CI)** | ***p*** | **AUC** | **ΔAUC** | **AIC** | **LR test p** |
| --- | --- | --- | --- | --- | --- | --- |
| Base model | — | — | 0.731 | Ref | 438.3 | Ref |
| ***CPR models*** | | | | | | |
| Binary CPR (< 5th centile) | 0.466 (0.278–0.780) | 0.004 | 0.749 | +0.018 | 431.4 | 0.003 |
| Continuous CPR | 2.100 (1.279–3.447) | 0.003 | 0.757 | +0.026 | 430.5 | 0.002 |
| Log-transformed CPR | 2.794 (1.487–5.247) | 0.001 | 0.761 | +0.030 | 429.7 | 0.001 |
| ***UA PI models*** | | | | | | |
| Binary UA PI (> 95th centile) | 0.705 (0.428–1.161) | 0.170 | 0.739 | +0.008 | 438.4 | 0.173 |
| Continuous UA PI | 0.306 (0.133–0.704) | 0.005 | 0.753 | +0.022 | 432.6 | 0.005 |
| Log-transformed UA PI | 0.244 (0.095–0.621) | 0.003 | 0.754 | +0.023 | 431.4 | 0.003 |
| ***MCA PI models*** | | | | | | |
| Binary MCA PI (< 5th centile) | 0.514 (0.296–0.892) | 0.018 | 0.754 | +0.023 | 434.9 | 0.020 |
| Continuous MCA PI | 1.639 (0.840–3.195) | 0.147 | 0.743 | +0.013 | 437.6 | 0.102 |
| Log-transformed MCA PI | 2.186 (0.901–5.304) | 0.084 | 0.744 | +0.014 | 437.1 | 0.075 |
| ***Combined UA PI + MCA PI models*** | | | | | | |
| Binary UA + binary MCA | See individual ORs | — | 0.756 | +0.025 | 435.9 | 0.041 |
| Continuous UA + continuous MCA | UA: 0.320 (0.140–0.735);  MCA: 1.549 (0.793–3.026) | UA: 0.007;  MCA: 0.200 | 0.761 | +0.030 | 432.4 | 0.007 |
| Log UA + log MCA | UA: 0.257 (0.101–0.652);  MCA: 2.041 (0.839–4.968) | UA: 0.004;  MCA: 0.116 | 0.763 | +0.032 | 430.8 | 0.003 |

**Abbreviations:** AIC, Akaike information criterion; AUC, area under the receiver operating characteristic curve; CI, confidence interval; CPR, cerebroplacental ratio; LR, likelihood ratio; MCA PI, middle cerebral artery pulsatility index; OR, odds ratio; UA PI, umbilical artery pulsatility index; ΔAUC, change in AUC relative to base model.

**Notes:** Base model includes nulliparity (OR 0.224, p < 0.001), pre-pregnancy BMI (OR 0.929 per kg/m², p < 0.001), and pre-gestational diabetes mellitus (OR 0.249, p = 0.009). Binary Doppler thresholds: UA PI > 95th centile (Acharya reference), MCA PI < 5th centile (Bahlmann reference), CPR < 5th centile (Ebbing reference). For binary UA and MCA, individual ORs are from the combined model (both entered simultaneously); AUC and LR test reflect the joint addition. LR test p-values are from likelihood ratio tests comparing each model to the base model. MCA PI distribution was markedly skewed (skewness 17.4, range 0.61–20.70) due to extreme outliers, rendering continuous and log-transformed MCA PI unreliable predictors despite adequate model convergence.
